# Supplementary material for: Fecal Microbial Structure and Metabolic Profile in Post-Weaning Diarrheic Piglets
Source: Genes (Basel). 2023 May 26;14(6):1166. doi: 10.3390/genes14061166 (PMC10298007; doi:10.3390/genes14061166)
Supplement: Supplementary file 1 [file genes-14-01166-s001.zip › Supplementary Figures.pdf]

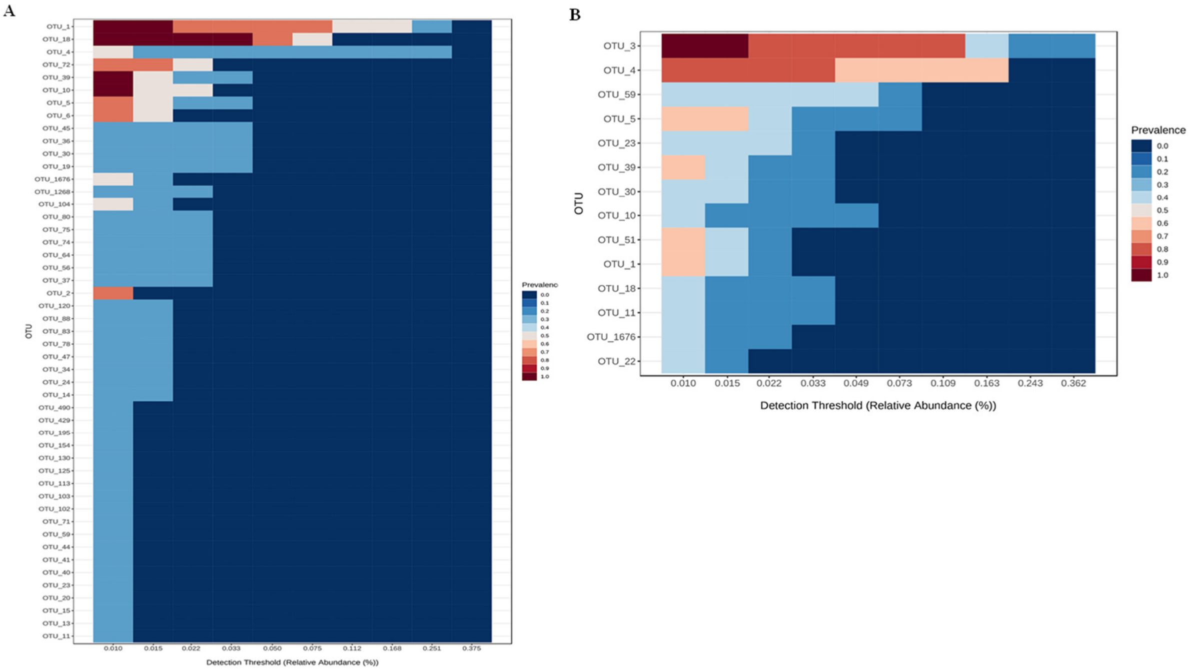

Figure S1. Core OTUs (with the relative abundance > 0.01%)in the feces of healthy (A) and diarrheic piglets (B).

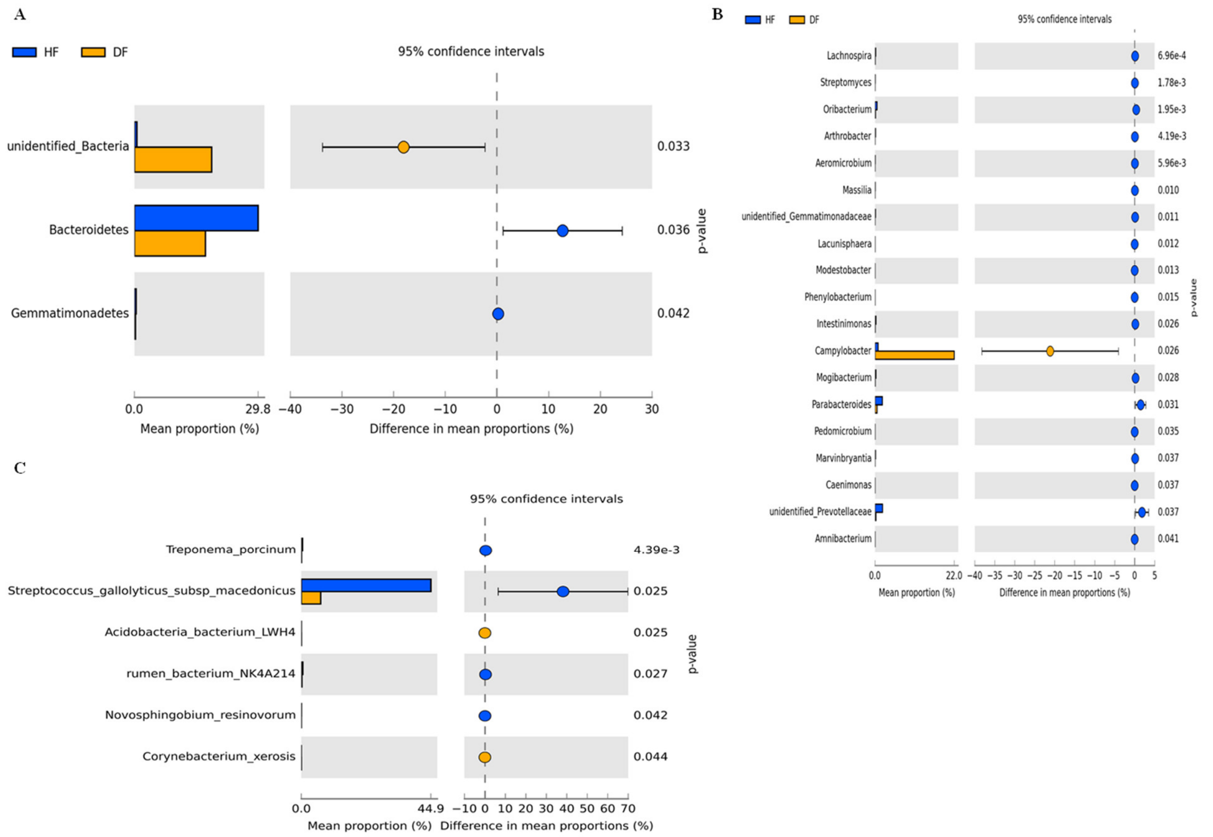

Figure S2. Differential microbiota between diarrheic and healthy piglets at the phylum (A), genus (B), and species (C) levels.

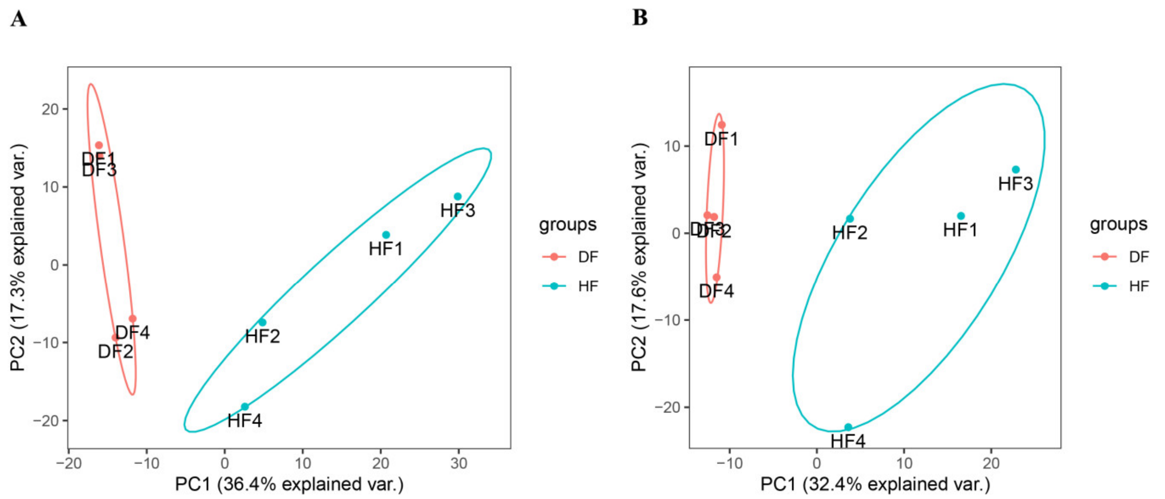

Figure S3. PCA analysis of total fecal samples in diarrheic and healthy piglets at positive (A) and negative (B) ions mode.

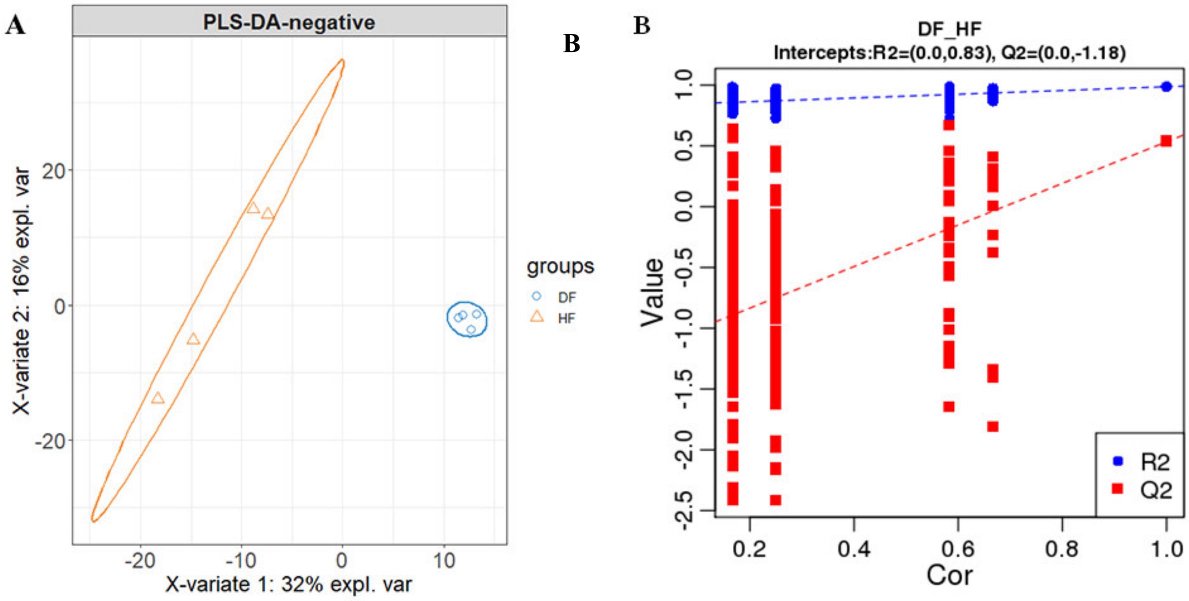

Figure S4. PLS-DA plot (A) and permutation test (B) derived from the fecal metabolite profiles of healthy and diarrheic piglets at negative ions mode.

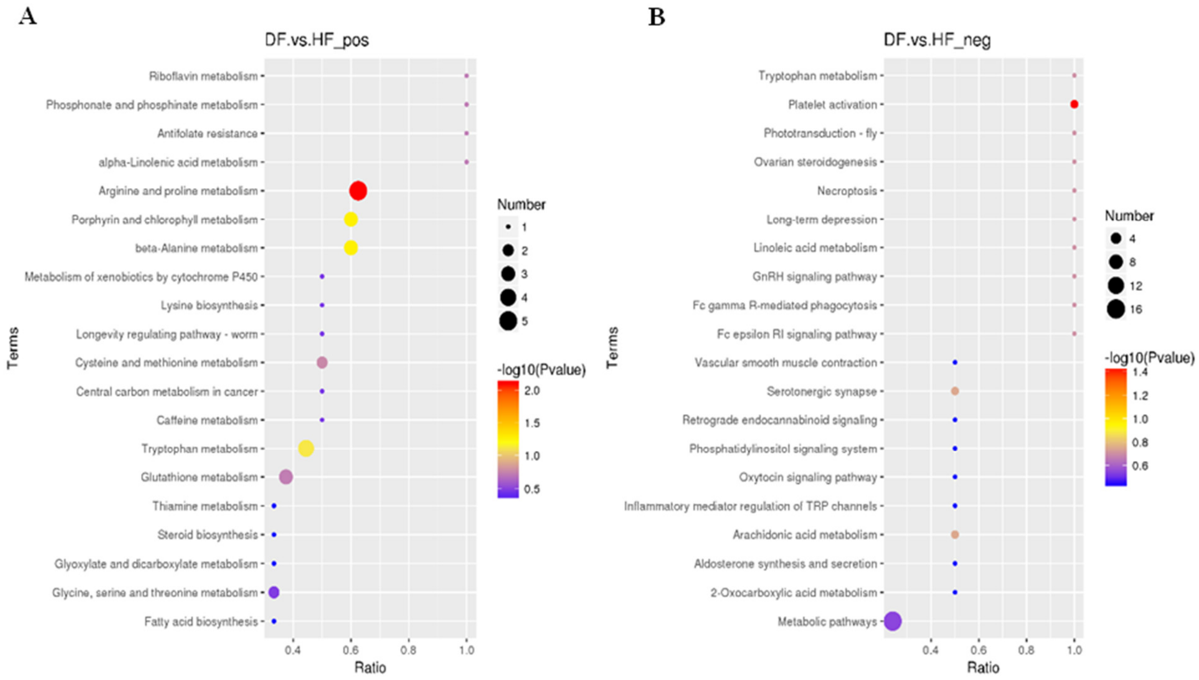

Figure S5. KEGG enrichment scatterplot of differential fecal metabolites at positive (A) and negative (B) ions mode.

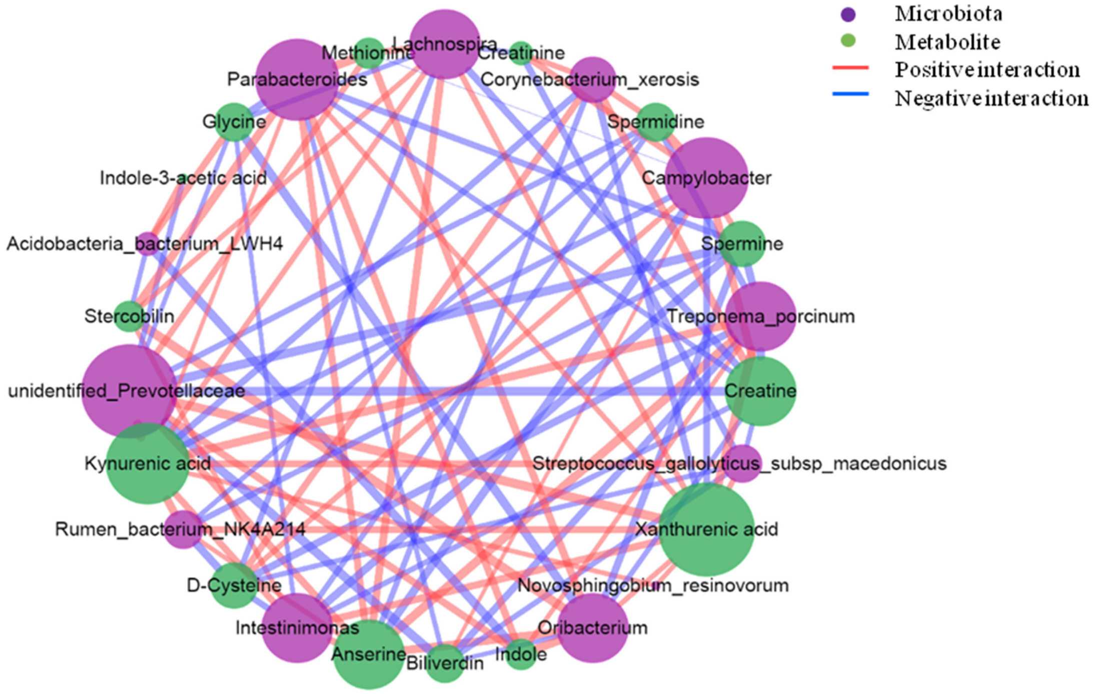

Figure S6. Correlation network of differential gut microbiota and fecal metabolites.
