# Supplementary material for: Fecal Microbial Structure and Metabolic Profile in Post-Weaning Diarrheic Piglets
Source: Genes (Basel). 2023 May 26;14(6):1166. doi: 10.3390/genes14061166 (PMC10298007; doi:10.3390/genes14061166)
Supplement: Supplementary file 1 [file genes-14-01166-s001.zip › Supplementary Table S1.pdf]

Table S1. Statistics of sample sequence number.

Group	Sample name	Raw PE	Raw Tags	Clean Tags	Effective Tags	Avg Len (bp)	Q20	Q30	GC%	Effective%
DF	DF1	92,090	85,670	81,474	67,486	413	96.76	90.68	51.69	73.28
	DF2	94,464	87,573	82,174	60,053	413	96.55	90.37	52.26	63.57
	DF3	97,754	91,468	85,789	62,936	414	96.46	90.18	51.36	64.38
	DF4	90,172	85,551	81,867	67,602	407	96.89	91.03	53.88	74.97
HF	HF1	98,643	91,612	85,472	63,868	421	96.15	89.57	53.34	64.75
	HF2	98,205	91,494	86,062	66,674	414	96.6	90.51	52.72	67.89
	HF3	97,003	90,036	83,811	64,953	419	96.25	89.84	53.59	66.96
	HF4	97,999	89,863	84,002	65,677	418	96.41	90.14	53.47	67.02

Raw PE: raw data from pair-end sequencing; Raw tags: the spliced raw data; Clean tags: the sequences after quality control, including filtrations of poor-quality bases and NA (not available) data. Effective tags: clean tags after removing the chimeric sequences; Avg Len: average length of total sequences. Q20: PHRED quality score threshold of 20; Q30: PHRED quality score threshold of 30; GC%: Guanine-Cytosine content; Effective%: the percentage of effective tags to raw PE data.
